# Molecular Epidemiological Investigations of Localized SARS-CoV-2 Outbreaks-Utility of Public Algorithms

**DOI:** 10.3390/epidemiologia3030031

**Published:** 2022-09-19

**Authors:** Mahmood Y. Bilal, James S. Klutts

**Affiliations:** 1 NOAH Clinical Laboratory, West Allis, WI 53214, USA; 2 SeqFORCE Consortium, Iowa City VA Health Care System, Iowa City, IA 52242, USA; 3 Department of Pathology, University of Iowa Hospitals and Clinics, Iowa City, IA 52242, USA

**Keywords:** SARS-CoV-2, disease outbreaks, RNA-seq, molecular epidemiology, Nextclade

## Abstract

The recent rapid expansion of targeted viral sequencing approaches in conjunction with available bioinformatics have provided an effective platform for studying severe acute respiratory syndrome coronavirus-2 (CoV-2) virions at the molecular level. These means can be adapted to the field of viral molecular epidemiology, wherein localized outbreak clusters can be evaluated and linked. To this end, we have integrated publicly available algorithms in conjunction with targeted RNASeq data in order to qualitatively evaluate similarity or dissimilarity between suspect outbreak strains from hospitals, or assisted living facilities. These tools include phylogenetic clustering and mutational analysis utilizing Nextclade and Ultrafast Sample placement on Existing tRee (UShER). We herein present these outbreak screening tools utilizing three case examples in the context of molecular epidemiology, along with limitations and potential future developments. We anticipate that these methods can be performed in clinical molecular laboratories equipped with CoV-2-sequencing technology.

## 1. Introduction

SARS-CoV-2 is part of the *Coronaviridae* family comprising positive-sense Ribonucleic Acid (RNA). Coronaviruses induce respiratory illnesses in infected individuals culminating as the common cold, pneumonia, or coronavirus disease-2019 (COVID-19) in the case of CoV-2 [1]. The transmission rate of CoV-2, which initiated in Wuhan/China in late 2019, had increased rapidly and concomitantly demanded an expansion in molecular diagnostics techniques for detection of virion RNA [1]. The basic detection methods operate on highly sensitive and specific quantitative reverse transcription PCR (qRT-PCR) assays [2]. These assays probe several locations across the CoV-2 RNA genome including Nucleocapsid, ORF1a/b, and Spike protein [1]. Upon the establishment of these PCR-based molecular assays across hospitals and laboratories worldwide, a secondary advanced molecular demand needed implementation. Research and clinical laboratories began using next-generation sequencing (NGS) techniques for typing CoV-2 variants. Specifically, targeted sequencing technologies for CoV-2 variant identification have expanded rapidly in 2022. This is at least partially due to the increased availability of user-friendly bench top sequencers, such as the Illumina MiSeq. This implementation was, in part, an attempt to grasp and elucidate the molecular dynamics of Cov-2 viral mutagenesis and dissemination. Iowa City Veterans Affairs (VA) laboratory, as part of the VA Sequencing for Research Clinical and Epidemiology (SeqFORCE) consortium, implemented sequencing technology for CoV-2 to assist treating physicians when clinically indicated. The latter includes vaccine breakthrough cases, reinfection with CoV-2, extended COVID-19 hospitalization, and local outbreak investigations. Outbreak investigations are typically two or more cases of a suspected intra-facility transmission event.

An epidemiological investigation for a transmissible infection consists of a multi-step plan that includes developing exposure data with respect to place, time, and persons involved. The goal is to ultimately trace back to the source (patient zero), identify other cases, control spread, and evaluate infection control procedures [3,4]. This type of investigation can be observed in the case of SARS-CoV-2 with a recent excellent report by Al Hamad et al. [5]. Their clinical investigation allowed tracing back to patient zero (index case), and re-evaluation of infection control procedures. While these investigations are important, as the authors mention, the addition of sequencing data would have provided more concrete evidence of strain sharing, at the molecular level [5].

Currently, there exist methods that utilize sequencing and epidemiological data in order to evaluate transmission dynamics. These include transphylo, scotti, and outbreaker2, along with other quantitative-based methods [6,7,8,9]. However, the workflow of some of these methods may not fit best in a clinical laboratory setting requiring personnel with complex computational or programming background. We herein agglomerate simple qualitative methods for determining molecular linkage in strains suspected to be part of a transmission ring. We present three case examples where methods involving Nextclade and UShER algorithms, which are publicly available for clinical laboratory personnel, were used [10,11,12]. Outbreak samples underwent phylogenetic clustering with locally sequenced samples utilizing Nextclade. Next, nucleotide mutational analysis was performed to determine specific shared regions that are carried through the strains of interest. The samples were then assessed via national databases (UShER) to determine global phylogenetic clustering and association with samples from similar regions. We believe that these methods are effective and practical due to their ease of use and convenience of publicly available software. These procedures can be utilized as part of a screening process for viral molecular epidemiological analysis in outbreak investigations. If needed, the investigator can then proceed to post-screening follow-up with statistical based algorithms [6,7,8,9].

## 2. Materials and Methods

### 2.1. Sample Collection and RNA Extraction

Viral Transport Media incubated with nasopharyngeal swabs were sent to our facility for CoV-2 sequencing. All samples were confirmed positive for CoV-2 RNA by the sending facilities through qRT-PCR. RNA extractions were performed using Qiagen’s *QIAamp Viral RNA kit*. A volume of 150 µL from Viral Transport Media (VTM) was combined with 560 µL of viral lysis buffer containing carrier RNA. The viral lysate was applied to Qiagen’s silica-based spin columns, and then centrifuged to bind viral RNA to the column. The column underwent multiple ethanol washes, and then the viral RNA was eluted in a volume of 45 µL of Qiagen’s AVE buffer. Exclusion criteria for any outbreak investigation were samples that had >30 cycle threshold (Ct) as defined by qRT-PCR. All outbreak CoV-2 sequences in this study were used by permission of originating facilities. Outbreak samples were either derived from assisted living, or hospital facilities which involved both patients and employees. No exclusions of samples were made on the basis of patient clinical history. All infections were deemed related at the time by hospital and regional epidemiologists with specific inquiry of whether the sequences are similar or not—regardless of the patients’ clinical history.

### 2.2. Next Generation Sequencing of CoV-2 Genome

Our laboratory’s targeted NGS for CoV-2 genomic analysis has been clinically validated for diagnostic purposes and was verified with the assistance of public health laboratories. Preparations of CoV-2 NGS libraries were performed with the *QIAseq SARS-CoV-2 Primer Panel*, as previously described [9]. Briefly, cDNA was made from 5 µL of RNA eluate, and then 2.5 µL of the cDNA reaction was amplified in a two-pool 30-cycle PCR reactions utilizing ARTIC v3 primers. After PCR purification with AMPure-XP paramagnetic beads, the DNA was diluted for subsequent fragmentation and Illumina adapter ligation reactions. Samples were then purified with magnetic beads, uniquely indexed with PCR reactions, and then underwent a final purification process. The latter PCR step allows the combination of multiple patient samples in one tube for subsequent sequencing. All single libraries were pooled and then loaded into Illumina’s reagent cartridge (300 cycle v2) on a standard flow cell at 7 pM. Sequencing quality controls, including cluster density, total reads, and percent reads reaching Q30, were all within optimal ranges provided by Illumina. In addition, secondary quality controls, provided by Illumina’s *DRAGEN COVID Lineage* software (v3.5.5), which reads and quantifies the sequencing files, were all within acceptable ranges. Attained depth and breadth of coverage were at least 500× and 95%, respectively.

### 2.3. Bioinformatics and Analysis of CoV-2 Variants

FASTQ files were obtained from the Illumina MiSeq. The files were uploaded onto Illumina’s (Illumina, 5200 Illumina Way, San Diego, CA, USA) DRAGEN COVID Lineage software (v3.5.5) was utilized to perform genome alignment, production of QC metrics, CoV-2 variant/clade identification, and FASTA consensus files.

### 2.4. Phylogenetic Clustering and Nucleotide Analysis of CoV-2 Genomes

The clustering of patient CoV-2 sequences locally and within global databases was performed by Nextclade’s algorithm (v1.14.1) https://clades.nextstrain.org/) [12] and UShER (https://genome.ucsc.edu/cgi-bin/hgPhyloPlace) [10]. Nextclade was also used for the determination of CoV-2 strain mutations in comparison to the original Wuhan strain. These algorithms require import of FASTA files that are generated by DRAGEN COVID Lineage software. Please refer to the discussion section of this manuscript for details on these algorithms.

### 2.5. Calculation of Nucleotide Mutation Frequency

*COVIDCG* (covidcg.org) was utilized for attaining frequency of mutational occurrence during time-periods (1 April 2021 to 14 December 2021) utilizing 1,127,754 CoV-2 sequences. Frequency is thence derived from the equation:

$$(\mathrm{Frequency}=\frac{\;\mathrm{Total}\;\mathrm{Occurence}\;\mathrm{of}\;\mathrm{Mutation}}{\mathrm{Number}\;\mathrm{of}\;\mathrm{CoV}-2\;\mathrm{Genomes}\;\mathrm{Assessed}}).$$

## 3. Defining Similarity/Dissimilarity of Outbreak Strains

The methodology used is composed of three categories integrated as part of the full molecular assessment of strains in question. The methods need not be done in order, but in general, we should expect harmonious findings from the three approaches towards either similarity or dissimilarity, especially within nucleotides of ORF1a. However, temporal dynamics of viral mutations may in some circumstances lead to less than 100% identity within the ORF1a gene in suspected strains [13,14]. Additionally, the unavailability of recently sequenced strains (i.e., close sampling dates within international databases) could lead to neutral or non-concordant results when performing global phylogenetic clustering. We will discuss these limitations in the following sub-sections.

### 3.1. Utility of Mutational Frequencies

It is important to define the prevalence of single nucleotide mutations for qualitative analysis of outbreak strains. Mutations with overly high frequencies (>65%) are relatively not as useful in terms of qualitatively linking suspect strains. In part, this is due to the decreased confidence of association when an already large number of circulating CoV-2 sequences carry these mutations. To this end, we noticed that absolute cut-offs of low vs. high mutational prevalence within CoV-2 are not clear within the literature. There were efforts by some groups who attempted to define the prevalence of mutations based on normalized frequencies in conjunction to viral temporal dynamics. For example, Arevalo et al. categorized CoV-2 mutations into low, medium and high prevalence for structural and non-structural proteins [13]. The authors studied 115 mutations that occurred above 3% prevalence. They presented high frequency mutations as nucleotide locations that do not show reduced changes in prevalence greater than 1% and instead increase rapidly. The low frequency mutations undulate temporally in prevalence but with an overall frequency below 15%. Based on this, we recommend that the choice of unique mutations within the outbreak samples should be based on mutations with less than 15% frequency and preference to frequencies of less than 3% and up to 10%. Next, due to temporal mutational dynamics we would expect a degree of variation within each mutation site [13,14]. Thus, frequency calculations should be dependent on recent circulating variants, not based on the bulk of total CoV-2 viral sequences. To this end, we found that *COVIDCG* software allows calculations of CoV-2 mutational frequencies based on set dates.

### 3.2. Method 1—Local Clustering

The goal of this step is to assess or screen for signs of identity within the outbreak strains when compared to other sequences obtained from same laboratory (i.e., local) utilizing Nextclade. In terms of conceptual probability, the higher number of non-outbreak samples used in this comparison will contribute to a general higher confidence in the screen. To this end, we recommend that the analysis should be performed in conjunction with 40–100 samples. This will allow formation of a “higher resolution” phylogenetic tree for better sample comparison. If the outbreak samples’ nucleotide sequence is drifting towards homology, then they will phylogenetically cluster together within the same branch or a close branch. In contrast, dissimilar samples will be dispersed at some level along the phylogenetic tree.

We suggest the 40–100 sequences used for comparisons should be composed of currently circulating strains with a close sampling date relative to the outbreak strains. This is because there is more confidence in strain association if the outbreak strains cluster together in the presence of samples containing similar homologous sequences (i.e., Delta strains with similar sampling dates). This is in contrast to observing clustering in outbreak strains in presence of older or different sequence pools that have not been as exposed to temporal dynamics of viral mutations (i.e., Delta strains previously sampled months apart). Evidence of temporally associated mutagenesis can in-part be observed from the expansion or splitting of the Delta strains into sub-Deltas, with more than 100 AY lineages (outbreak.info).

Another consideration for this method is that its strengths are exhibited when comparing similar clades, such as Delta to Delta. When the outbreak strains are of different clades (i.e., Alpha or Omicron) it would be already highly demonstrative for association without clustering given the main circulating pool of variants at the time of this writing is Delta. In this case, mutational analysis (method-2) should be performed to probe homogeneity of CoV-2 genomic regions in the outbreak samples.

### 3.3. Method 2—Analysis of Genomic Mutations

In this step, we show the use of Nextclade nucleotide sequence alignment interface. The tool is used to infer signature mutations that are carried through the strains of interests, in comparison to other strains derived from alternate sources. Several genomic regions could be utilized for this goal. We found that, within the same clade, ORF1a is relatively unstable in genetic consensus (i.e., more informative) where one may observe unique/shared mutations only within the outbreak strains. This is in contrast to the Spike region, where it was not useful (i.e., does not change) when comparing strains from a matching clade. Similar to phylogenetic clustering, there is more confidence when detecting peculiar set of mutations in outbreak samples in presence of non-related samples with relatively homologous sequences (i.e., Delta strains with similar sampling dates). If the strains of interest are of different clade in the presence of a circulating dominant strain, then this would be already indicative of association. Nevertheless, mutational analysis should be performed to confirm identity.

A limitation with the use of molecular analysis is locating unique mutations with low frequency within the global CoV-2 sequence pool (<0.15 frequency “<15%”). We found that *COVIDCG* provides GISAID-based data with options to designate timeframes for attaining mutational frequencies. *COVIDCG* data, as well as Nextclade alignment, show that the Delta variant carries high frequency mutations in ORF1a. An example would be G4181T or C6402T, which are present at a frequency of more than 0.65 and should be excluded from comparisons. In some cases, however, the absence of high-frequency mutations may be useful for defining associations. The observation of 1–5 low-frequency shared mutations in outbreak strains is indicative of association. On the other hand, inability to find unique mutations does not exclude association or linear viral transmission. To this end, these genomic regions should be labeled as “non-informative” and attempts to analyze other regions should be performed.

Another limitation is that one should not expect that all outbreak samples to be identical within the ORF1a gene (see case-2). It should be expected that the longer the transmission chain, the more likelihood of virions drifting towards dissimilarity. In this case, the choice of shared low mutational frequencies will produce higher confidence in association–even if some of the samples are not fully identical. As a troubleshooting step, one can compare sampling dates between outbreak strains to determine if one or two, not fully identical strains, were sampled more than one week apart in a long outbreak chain. If this is the case, then the minor differences can be attributed to viral temporal mutational dynamics.

### 3.4. Method 3–Global Clustering (UShER)

This method is similar to local clustering by Nextclade; it produces a secondary layer of confirmation in strain-association. First, if samples are genetically similar or identical, they will cluster together in the presence of a global CoV-2 sequence base. Second, we have observed that outbreak CoV-2 sequences will tend to home or co-cluster near other samples sequenced from the same sending state. The latter would be indicative of association and possibly link to other related small sub-outbreaks occurring within these regions. A limitation is that homing to state of origin is dependent on available recent CoV-2 sequences from that region in order to compare to samples in-question. Therefore, not observing co-clustering with samples from the same sending state should not be taken as evidence of dissimilarity. Instead, overall results should be integrated from all three methods.

## 4. Outbreak Case Examples

In this section we will present three cases wherein two evinced similar or identical outbreak strains and one displayed indicators of dissimilar properties.

### 4.1. Case 1

Our laboratory received 12 viral specimens for evaluation of CoV-2 strain identity in a suspected outbreak. The outbreak occurred within a hospital facility involving patients and staff members. Two of the samples did not contain enough viral RNA due to high qRT-PCR Ct values (38–41 Ct)–thus no informative genomic analysis could be derived from these two samples. The other ten samples were all typed as sub-Delta variant AY.44. This was highly indicative of similarity between all ten strains given that the Delta variant had been split into more than 100 sub-strains at the time of this writing. Demonstration of only one strain in ten suspect specimens substantially increases the likelihood that an identical strain is the cause of this outbreak.

**Figure 1:**
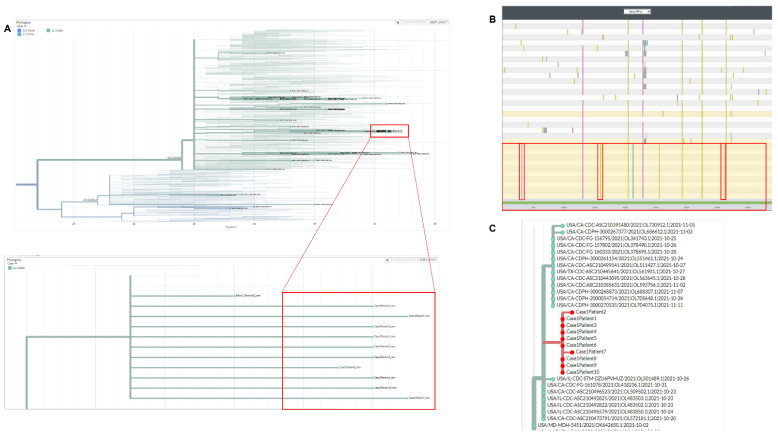
Phylogenetic and molecular analysis for outbreak case-1. (**A**) Nextclade phylogenetic clustering (cases highlighted by red boxes). (**B**) Nextclade gene viewer for CoV-2 ORF1a. Unique mutations are highlighted in red. (**C**) UShER analysis/clustering for outbreak samples with placement near most similar CoV-2 sequences.

To understand the shared AY.44 strain at the molecular level, the ten outbreak isolates were allowed to phylogenetically cluster based on genomic nucleotide similarities in comparison to 47 strains derived from other regions. All outbreak samples clustered together within the 21J Delta clade (Figure 1A). Nextclade nucleotide alignment system was then used to analyze ORF1a gene of CoV-2 within the ten strains in-question in comparison to other non-related Delta-isolates. We found that the outbreak strains were identical at ORF1a (Figure 1B). The ten strains carried three mutations unique to the isolates (Figure 1B–red bars). *COVIDCG* showed that the unique mutations C1191A/C5055T/C11109T had frequencies of 0.000641/0.000727/0.00156, respectively. There were other mutations that were not unique to the outbreak samples, but nevertheless appeared at a lower frequency (appeared in 1–2 other non-outbreak samples). An example of the latter is C6638T with frequency of 0.0637 or C7926T with frequency of 0.123. Nucleotide identity at ORF1a along with the presence of unique/shared low frequency mutations within all ten isolates suggest that all patients share the same unique strain.

Next, we evaluated the ten samples utilizing the national database, UShER [10]. This allows placement of the strains in question amongst other global sequences that are most related. We first observed that all ten outbreak strains clustered together, indicating they are all relatively homologous in overall nucleotide sequence (Figure 1C). Second, all outbreak strains aligned with other samples sequenced from same state (Illinois) that had a close sampling time. Overall, given the timing and location of the ten specimens we concluded the presence of evidence towards a localized transmission chain within the hospital facility as demonstrated by molecular sequencing analysis.

### 4.2. Case 2

**Figure 2:**
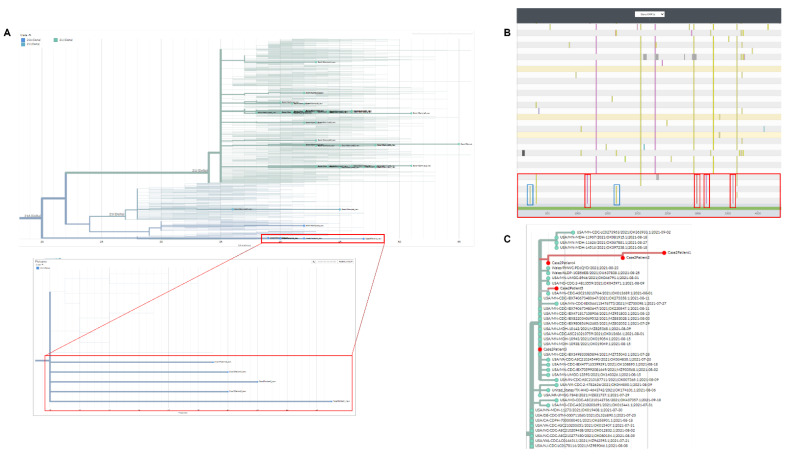
Phylogenetic and molecular analysis for outbreak case-2. (**A**) Nextclade phylogenetic clustering (cases highlighted by red boxes). (**B**) Nextclade gene viewer for CoV-2 ORF1a. Unique mutations are highlighted in red. (**C**) UShER analysis/clustering for outbreak samples with placement near most similar CoV-2 sequences.

Five suspect outbreak specimens were sampled at an assisted-living facility and then sent to our laboratory for molecular analysis. All five samples were typed as Delta variant B.1.617.2. The outbreak strains phylogenetically clustered together in exclusion of other Delta strains sampled at other locations (Figure 2A). Nextclade displayed four unique mutations that were not present in other Delta strains (Figure 2B–red bars). The mutations were C3737T/G9203A/T9678C/C11005A with a frequency of 0.00113/0.00951/0.00917/0.00976, respectively. Interestingly, three of five outbreak samples contained unique mutations in exclusion of the other two (Figure 2B–blue bars). Sampling dates for these isolates did not suggest temporally dependent mutations as four were sampled on the same date, and one three days later. Therefore, the simplest explanation would be random mutations occurring in the middle of transmission and then carried over to the last sampled specimen. The latter would be in conjunction with alternate sampling during low to peak infection cycles when the virion had replicated exponentially within the host [14]. In this case, sampling the first two patients at initial infection cycle, followed by the last three at peak infection cycle could explain the differences in mutations. We did not have clinical information on the suspected transmission sequence or if there was a patient “zero” within these samples. Next, UShER analysis placed the five samples on the same phylogenetic location along with other sequences from the same state sampled at around the same timeframe (Minnesota–Figure 2C). Given the common source of samples, timing of outbreak and signature low frequency mutations, we concluded that these patients shared the same strain of the Delta variant.

### 4.3. Case 3

**Figure 3:**
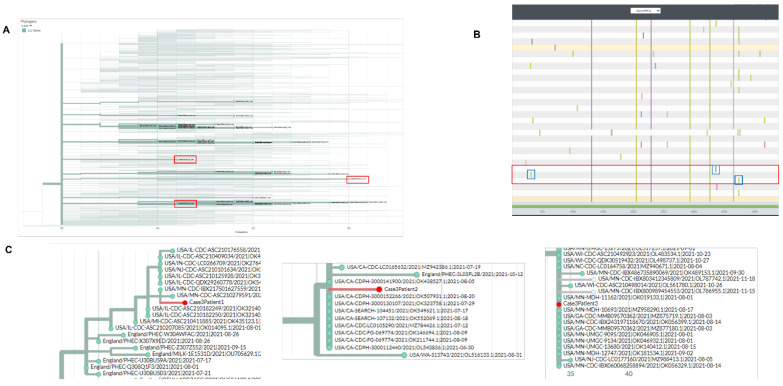
Phylogenetic and molecular analysis for outbreak case-3. (**A**) Nextclade phylogenetic clustering (cases highlighted by red boxes). (**B**) Nextclade gene viewer for CoV-2 ORF1a. Unique mutations are highlighted in red. (**C**) UShER analysis/clustering for outbreak samples with placement near most similar CoV-2 sequences.

Three CoV-2 specimens were sent for analysis of genetic similarity in a suspected hospital facility outbreak. Although all three samples were of the 21J Delta clade, they had instead typed as different Delta sub-variants (B.1.617.2, AY.3, and AY.36). This was a first indicative for non-similarities of these strains. Local phylogenetic clustering compared to strains from other locations showed that the three strains dispersed away from each-a second indication of non-similarity (Figure 3A). Nextclade nucleotide alignment system was used to analyze the ORF1a gene of CoV-2 (Figure 3B). Although the samples had similar ORF1a signature, they were comparable to all other Delta variants analyzed. The reason is that the three suspect strains carried mutations that occurred at high frequency such as G4181T (0.685 frequency) and C7124T (0.670 frequency). Therefore, they could not be used for defining uniquely carried mutations within the three strains. Importantly, each suspect strain carried a mutation that was not present on the other two suspect strains (Figure 3B–blue bars). UShER phylogenetic cluster analysis placed the three strains in three different clusters that were associated with sequences from different states (Figure 3C). We concluded from this analysis that these suspect strains are not identical.

## 5. Discussion

In this manuscript, we described the utility of publicly available algorithms in order to assist epidemiological investigations. Here, molecular analysis of CoV-2 outbreak sequences can be assessed at multiple levels (i.e., local/global phylogenetic clustering, and specific mutational analysis). This process yields a three-layer qualitative analysis that, in many cases, produces confident results in terms of defining strain similarity/dissimilarity.

It is important to note that, similar to other diagnostic laboratory tests, these methods presented here should only serve as an outbreak screen in-part due to limitations mentioned above. For example, one cannot produce a quantitative estimate for strain relatedness since no direct statistical comparison is involved other than qualitative grouping of the samples based on genetic similarities (NextClade, UShER) [10,11,12]. In this case, the clinical laboratory may need to extend their findings to other available methodologies involving statistical computing [8,9]. The latter includes transphylo, scotti, outbreaker2, and other quantitative-based methods [6,7,8,9]. However, while these methods can provide a quantitative evaluation, expertise in computational methods and/or R-programming is required.

Another limitation is the focused use of Cov-2 ORF1a region. We have found that the SARS-CoV-2 ORF1a region contains a degree of nucleotide instability (i.e., more informative) when compared to the Spike region. This can be true even between strains that are of the same sub-lineage–which has been observed for both Delta and Omicron strains. Regions, such as ORF1a, with relative genomic instabilities can be utilized to compare outbreak strains qualitatively by using low frequency mutations as a first-tier choice. However, we have found that sometimes, other regions may assist in identifying group similarities in the case where the ORF1a region is identical between all samples (e.g., Gene M). We refer the reader to a discussion on the subject of ORF1a use [9].

Care should be taken when evaluating mutational frequencies using *COVIDCG* or any other frequency-producing software [15]. We have initially hypothesized, and confirmed through our outbreak analyses, that mutational frequencies for outbreak samples will drift temporally. The temporal drift is observed even within the same CoV-2 lineages. Therefore, mutational frequencies must be based on the specific outbreak period. For example, if an outbreak had occurred in November of 2021, then compiling or averaging mutational frequencies from January 2021–September 2021 may not produce accurate ORF1a frequencies. In fact, in some cases it can be misleading wherein a true rare mutation would be considered common, or a true common mutation would be considered rare. Therefore, for the November 2021 example, a better *COVIDCG* date range would be August 2021–November 2021. Narrower date ranges provide more accurate frequencies that better approach true values of circulating variants, at the time of the outbreak. However, a narrower date range will contain a lower sample number. Therefore, we advise the outbreak investigator to compare both narrow (October 2021–November 2021) as well as slightly larger range (August 2021–November 2021). In this case, if the two ranges provide similar numbers, then use of the wider date range is recommended as it contains a higher sample number. Overall, since the methods mentioned here are only qualitative, slight deviation in frequencies will not affect the final outcome of the investigation.

In conclusion, we propose that along with qualitative analysis, clinical laboratories should consider quantitative probability-based models to estimate the likelihood or relatedness of outbreak strain associations. This model should include inputs for outbreak sample number, mutational frequencies, and potentially spatiotemporal dynamics. Overall, the above methods can function as a platform for future refinements and developments but may also be used as is to define strain association in localized viral outbreaks.

## Data Availability

The molecular data presented in this study are available upon request from the corresponding author.
